# Perforated small bowel lymphoma: a rare presentation of Crohn’s disease

**DOI:** 10.1093/jscr/rjae135

**Published:** 2024-03-11

**Authors:** Abdalrahman N Herbawi, Osama Hroub, Omar H Salloum, Kareem Ibraheem, Qusai A Alsalah, Ahmad G Hammouri, Rafiq Salhab

**Affiliations:** Faculty of Medicine, Palestine Polytechnic University, Hebron 9020000, Palestine; Faculty of Medicine, Palestine Polytechnic University, Hebron 9020000, Palestine; Faculty of Medicine, Palestine Polytechnic University, Hebron 9020000, Palestine; Faculty of Medicine, Palestine Polytechnic University, Hebron 9020000, Palestine; Faculty of Medicine, Palestine Polytechnic University, Hebron 9020000, Palestine; Radiology Department, Al-Ahli Hospital, Hebron 9020000, Palestine; Faculty of Medicine, Palestine Polytechnic University, Hebron 9020000, Palestine; General Surgery Department, Al-Ahli Hospital, Hebron 9020000, Palestine

**Keywords:** Crohn's disease, non-Hodgkin's lymphoma, immunosuppressive therapy, small bowel perforation, diffuse large B-cell lymphoma

## Abstract

Adenocarcinoma and lymphoma, potential complications of Crohn's disease (CD), may result in small intestinal perforations, particularly in those on immunosuppressive therapy. The ileum is typically the site of small intestinal perforations in CD, and the link between CD and lymphoma remains uncertain. This case report explores a long-term CD patient on immunosuppressive therapy who presented with acute abdominal pain. Imaging revealed signs of intestinal perforation, successfully managed with surgery. The final pathology report confirms the diagnosis of diffuse large B-cell lymphoma. This report sheds light on the complicated nature of gastrointestinal lymphoma in CD patients.

## Introduction

Malignant lymphoma of the gastrointestinal tract is a rare condition; in most cases, it takes place within the stomach and can affect the colon or small intestine [1]. About 70% of Crohn's disease (CD) patients will need surgery to help ease their symptoms, even with advanced medical treatments [2]. Patients with inflammatory bowel disease (IBD), especially those undergoing immunosuppressive therapy, have shown a higher occurrence of non-Hodgkin's lymphoma [1]. Perforations in the jejunum and ileum may develop simultaneously, or they may develop frequently as separate perforations throughout the long-term clinical course of CD [3]. When CD is present, the ileum is typically the site of small intestinal perforations. However, in rare cases, nearby locations like the jejunum may also be impacted [4]. In cases of CD, complications can arise in the form of malignant adenocarcinoma or lymphoma, which can manifest as small bowel perforation [5]. Also, the reason behind the observed connection between CD and lymphoma remains unclear [1]. Herein, we present a 47-year-old woman with a history of CD who presented with acute abdominal pain. Exploratory laparotomy revealed a perforation in the jejunum and abnormal wall thickening in the ileum, diagnosed as diffuse large B-cell lymphoma (DLBCL) based on the pathology report after surgery.

## Case presentation

A 47-year-old woman with a 12-year history of CD presented with abdominal pain. She had been intermittently treated with azathioprine; she had been hospitalized for multiple relapses for exacerbations of CD; and her management was conservative. The patient had an episode of perforation 7 years ago located in the terminal ileum as a complication of CD, and she underwent laparotomy resection and anastomosis to the site of perforation.

**Figure 1:**
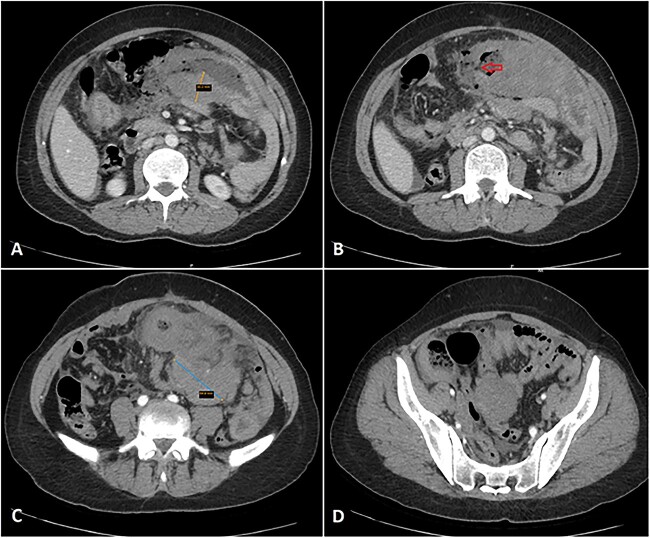
Multiple axial cuts of the patient’s abdomen CT scan with IV contrast are shown. Extensive bowel wall thickening up to 3 cm is noted, involving a long segment of small bowel loops in the left abdomen (jejunal loops) with a gray pattern of enhancement (A). Additionally, a wall defect/perforation is seen with the surrounding pneumoperitoneum and spillage of fecal material within the peritoneal cavity (B). Associated multiple enlarged adjacent mesenteric lymph nodes are also demonstrated, forming a large mass-like lesion with surrounding fat stranding (C). Moreover, mild wall thickening involving another short segment of small bowel loops in the right abdomen (ileal loops) is also noted (D).

The patient presented to the emergency department with 2 h of sudden-onset abdominal pain, vomiting, diarrhea, nausea, and loss of appetite. Her vital signs were a blood pressure of 126/78 mmHg, a pulse of 107 bpm, and a temperature of 38.8°C. Abdominal examination found diffuse tenderness and distinction with a positive rebound test. The laboratory tests showed abnormally elevated inflammatory markers (CRP = 247 mg/L, WBC = 9500 cells/mm^3^, PMNs = 88%). An abdominal contrast computed tomography imaging showed wall thickening of the jejunum, ileum, and adjacent mesenteric lymphadenopathy, suggesting a Crohn’s flare (Fig. 1).

**Figure 2:**
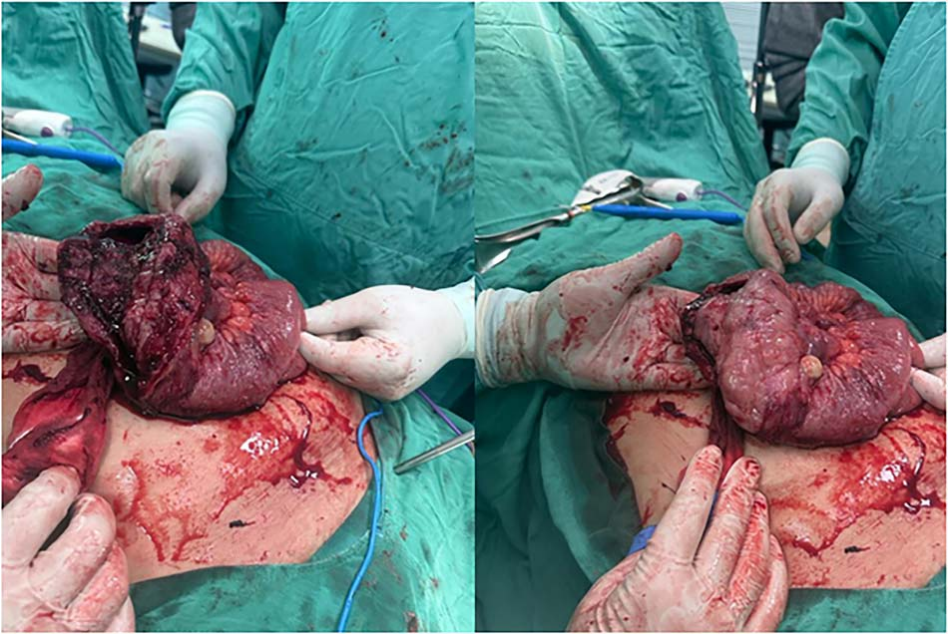
Intraoperative view of perforated small bowel tumor.

An exploratory laparotomy was done with a provisional diagnosis of viscus perforation. A midline incision was given, and the abdomen was opened in layers. We found an enlarged and dilated segment of the jejunum, ~30 cm in length, located 50 cm from the ligament of Treitz, with a perforation of about 8 cm. Additionally, we observed an enlarged and dilated segment of the ileum, ~10 cm in length, situated 30 cm from the ileocecal valve (Fig. 2). The jejunum and ileum segments were extracted and subjected to end-to-end anastomosis. A gross biopsy was sent to histopathology, revealing DLBCL with positive CD20, CD10, and BCL5 immunohistochemistry.

## Discussion

IBD patients treated with azathioprine/6-MP face a fourfold higher risk of lymphoma. This elevated risk may stem from the medications, the severity of the underlying disease, or a combination of both factors [5]. From a histological perspective, diverse subtypes characterize small bowel tumors. Adenocarcinoma is the most common type, accounting for 47% of cases, trailed by carcinoid tumors at 28%, whereas GI lymphomas and GI sarcomas each make up 12% of the cases [6].

Instead of a distinct inclination for spontaneous free perforation development at a specific site in CD, possibly linked to the thinner wall of the ileum when compared with the jejunum, the prevalence of jejunal and ileal perforations is more likely a reflection of the general distribution of the disease across different sites within the small intestine. This indicates a higher involvement of the ileum compared with the proximal part of the small intestine [3].

The perforation rate in gastrointestinal lymphoma is about 9%–22%, exceeding the rate observed in high-grade lymphomas such as DLBCL [7]. DLBCL represents a fast-growing B-cell lymphoma, histologically identified by the widespread growth of large cancerous B lymphoid cells whose nucleus size matches or surpasses the nuclei of typical histiocytes [8]. In cases of CD, there is a potential for complications such as malignant adenocarcinoma or lymphoma, which can lead to small bowel perforation [1].

Primary gastrointestinal lymphoma can lead to a reduction in the mechanical integrity of the gastrointestinal wall, potentially resulting in intestinal perforation [7]. Several studies have indicated that perforation is an unfavorable prognostic indicator for individuals with high-grade lymphomas such as DLBCL [3].

## Conclusion

In this rare case, a 47-year-old woman with longstanding CD and azathioprine treatment presented with acute abdominal pain. Exploratory laparotomy revealed a perforation in the jejunum and abnormal wall thickening in the ileum, diagnosed as DLBCL. The complex interplay between CD, immunosuppression, and lymphoma highlights diagnostic challenges. Elevated lymphoma risk in CD patients adds complexity, whereas the diverse histological subtypes and atypical perforation sites emphasize the need for tailored management and further research.

## Data Availability

The data used to support the findings of this study are included in the article.
