# Correction to: Perforated small bowel lymphoma: a rare presentation of Crohn’s disease

**DOI:** 10.1093/jscr/rjae237

**Published:** 2024-07-12

This is a correction to: Abdalrahman N Herbawi, Osama Hroub, Omar H Salloum, Kareem Ibraheem, Qusai A Alsalah, Ahmad G Hammouri, Rafiq Salhab, Perforated small bowel lymphoma: a rare presentation of Crohn's disease, *Journal of Surgical Case Reports*, Volume 2024, Issue 3, March 2024, rjae135, https://doi.org/10.1093/jscr/rjae135.

In the originally published version of this manuscript, the contact address for the corresponding author was listed incorrectly as: General Surgery Department, Al-Ahli Hospital, Hebron 9020000, Palestine. This has been corrected to read: Faculty of Medicine, Palestine Polytechnic University, Hebron 9020000, Palestine.

